# Real-World Effectiveness of Dalbavancin in Osteomyelitis Without Implantable Devices: A Retrospective Monocentric Study

**DOI:** 10.3390/antibiotics15070640

**Published:** 2026-06-26

**Authors:** Giorgio Tiecco, Angelica Lenzi, Federico Cesanelli, Evelyn Van Hauwermeiren, Francesco Rossini, Alessio Sollima, Alice Mulé, Silvia Lorenzotti, Liana Signorini, Francesco Castelli, Eugenia Quiros-Roldan

**Affiliations:** 1 Department of Clinical and Experimental Sciences, Unit of Infectious and Tropical Diseases, University of Brescia and ASST Spedali Civili di Brescia, 25123 Brescia, Italy; g.tiecco@unibs.it (G.T.); a.lenzi001@unibs.it (A.L.); f.cesanelli@unibs.it (F.C.); f.rossini009@unibs.it (F.R.); a.sollima@unibs.it (A.S.); francesco.castelli@unibs.it (F.C.); 2 Unit of Infectious and Tropical Diseases, ASST Spedali Civili di Brescia, 25123 Brescia, Italy; efu@hotmail.it (E.V.H.); silvia.lorenzotti@libero.it (S.L.); liana.signorini@unibs.it (L.S.); 3 Department of Global Public Health, Karolinska Institute, 17177 Stockholm, Sweden

**Keywords:** dalbavancin, osteomyelitis, off-label, implantable devices, Gram positive

## Abstract

Background: Dalbavancin (DBV) is a long-acting lipoglycopeptide with activity against Gram-positive pathogens approved for the treatment of acute bacterial skin and skin structure infections (ABSSSI). Its pharmacological profile supports use in infections requiring prolonged therapy, yet its role in osteomyelitis without implantable devices (OM-WoID) remains off-label. This study aims to describe real-world DBV use in a large tertiary care hospital, focusing on its effectiveness in OM-WoID. Methods: This is a monocentric, retrospective analysis including all patients receiving DBV at ASST Spedali Civili di Brescia, Italy, from April 2017 to July 2023. The statistical analysis focused on patients who received DBV for either ABSSSI or OM-WoID, with the latter transitioning to DBV after traditional daily intravenous therapy. Clinical, microbiological, and treatment data were extracted from electronic records and stored in REDCap. Effectiveness was defined as infection resolution or improvement; treatment failure encompassed clinical worsening, recurrence or suppressive therapy. Predictors of failure were identified through univariate and stepwise multivariate logistic regression. Results: During the study period, 157 patients (63.0% male; mean age 62.5 ± 20 years) received at least one dose of DBV, predominantly for off-label indications (66.2%). Early discharge was the most common reason for switching to DBV (66.3%). Focusing specifically on patients treated for ABSSSI (53) and OM-WoID (43), treatment success was achieved in 81.1% of ABSSSI and 90.7% of OM-WoID cases. In the stepwise multivariate logistic regression, older age was independently associated with an increased risk of treatment failure (OR 1.07, 95% CI 1.01–1.13; *p* = 0.028), while the presence of multimorbidity significantly reduced the risk (OR 0.07, 95% CI 0.01–0.77; *p* = 0.029). Discussion: Our study offers a comprehensive real-world analysis of DBV use in both approved and off-label indications. Although current clinical experience with DBV remains limited, DBV emerges as a valuable step-down option for the management of invasive Gram-positive infections in our setting. Consistent with previous evidence, older age independently increased the risk of treatment failure, whereas multimorbidity appeared protective, likely due to selection bias and the more intensive monitoring, earlier interventions, and tailored management such patients often receive. Our results support a broader range of approved indications for DBV to allow earlier discharge and more efficient use of healthcare resources.

## 1. Background

Dalbavancin (DBV) is a long-acting lipoglycopeptide antibiotic approved for the treatment of acute bacterial skin and skin structure infections (ABSSSI), administered either as a single 1500 mg dose or as a 1000 mg dose followed by 500 mg one week later via a 30 min intravenous infusion [1]. It exhibits in vitro activity against a broad range of Gram-positive microorganisms including *Staphylococcus aureus*, coagulase-negative staphylococci (CoNS), enterococci, and streptococci [2]. Moreover, DBV remains effective against multidrug-resistant (MDR) pathogens, including methicillin-resistant *S. aureus* (MRSA) and non-VanA vancomycin-resistant enterococci, making it a valuable option for challenging infections [1]. As a bactericidal agent, DBV inhibits bacterial cell wall biosynthesis and is characterized by a long terminal half-life, high concentrations in several tissues, and a favorable safety profile [2].

Key clinical trials have established dalbavancin as a non-inferior agent to vancomycin and linezolid for ABSSSI, including those caused by MRSA. The DISCOVER 1 and 2 trials demonstrated that once-weekly dalbavancin (two-dose regimen: 1000 mg IV day 1, 500 mg IV day 8) was non-inferior to daily vancomycin (with optional switch to oral linezolid) in early clinical response and overall clinical success, with fewer adverse events and greater dosing convenience [3]. A single-dose regimen (1500 mg IV) was also shown to be non-inferior to the two-dose regimen, with similar safety and efficacy [4]. Comparative studies and meta-analyses confirm that dalbavancin’s efficacy is statistically similar to vancomycin, teicoplanin, linezolid, and daptomycin for ABSSSI and MRSA infections, with a lower likelihood of adverse events compared to vancomycin and linezolid [5,6,7,8]. Dalbavancin’s long half-life allows for single or weekly dosing, which is a major advantage over daily dosing required for vancomycin, teicoplanin, linezolid, and daptomycin, improving outpatient management and reducing hospital stay [3,4,9,10]. Recent randomized trials in *S. aureus* bacteremia (including MRSA) show dalbavancin is non-inferior to standard therapy (vancomycin or daptomycin) for clinical efficacy, but not superior in composite outcomes; safety profiles are comparable, with fewer catheter-related complications due to reduced need for prolonged IV access [11].

Given its pharmacological properties, DBV has recently gained attention as a potential therapeutic option for various infections, particularly those requiring prolonged antibiotic treatment, such as bone and joint infections or endocarditis [12,13,14]. Its off-label use has been increasingly reported in clinical practice, highlighting its potential role in sequential therapy for these conditions [1]. Notably, DBV has been associated with a reduction in hospital length of stay, decreased treatment-related costs, and improved patient quality of life, underscoring its pharmacoeconomic benefits [15]. While DBV shows promise in the management of osteomyelitis from clinical, microbiological, pharmacological, and economic perspectives, its use in this setting remains off-label and not yet formally established [1].

Dalbavancin is best positioned as an alternative to conventional anti-MRSA agents for ABSSSI and select invasive infections when outpatient therapy, reduced hospital stays, or avoidance of long-term IV access is desired. It is not first-line for severe, deep-seated infections (e.g., left-sided endocarditis) but is increasingly used for consolidation therapy in bacteremia and right-sided endocarditis after initial clearance [4,12,16]. Its dosing convenience and safety profile make it a valuable option in the anti-MRSA armamentarium, especially for patients where adherence or IV access is problematic.

This retrospective monocentric study describes the use of DBV at ASST Spedali Civili di Brescia, Italy, with a particular focus on its effectiveness in osteomyelitis without implantable devices (OM-WoID), thereby contributing to the growing body of evidence supporting its clinical application in this difficult-to-manage setting.

## 2. Results

**Figure 1:**
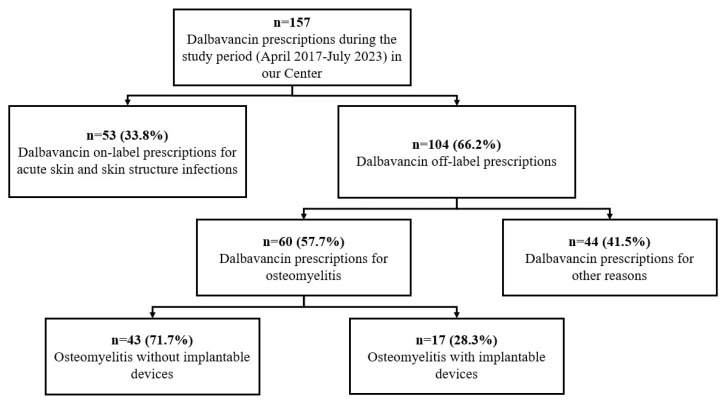
Flow chart illustrating the patient recruitment and selection process during the study period.

During the study period, at least one dose of DBV was administered, either on-label or off-label, to 157 patients. The patient recruitment and selection process is summarized in the flow chart presented in Figure 1.

Most patients were male (63.0%) with a mean age of 62.5 years (±20). Most patients were admitted to medical wards (80.9%), and 88.5% presented with at least one comorbidity, the most frequent being cardiovascular diseases (54.6%) and diabetes (28.0%). DBV was prescribed for off-label indications in 66.2% of patients, with osteoarticular infections—including OM-WoID (20.4%) and prosthetic joint infections (7.6%)—representing the most common off-label uses. Microbiological diagnosis was predominantly monomicrobial (81.5%), with methicillin-resistant *Staphylococcus aureus* (MRSA) and methicillin-resistant coagulase-negative staphylococci (MR-CoNS) being the most frequently isolated pathogens. Early discharge was the most common reason for switching to DBV (66.3%), and overall clinical improvement or recovery was achieved in 77.1% of cases, with minimal adverse events reported. All the results are extensively shown in Table 1.

We focused on the two largest patient groups receiving DBV, namely those treated for ABSSSI and OM-WoID: we analyzed a total of 96 cases, comprising 53 ABSSSI and 43 OM-WoID (Table 2). Demographic variables were comparable between the groups, with a median age of 63 years (range 10–91 years) in ABSSSI and 70 years (range 9–92 years) in OM-WoID, and a predominance of males in the OM-WoID group (67.4% vs. 49.1%). CA infections were more frequent in ABSSSI (64.2%), whereas HCA infections predominated in OM-WoID (46.5%). Microbiological documentation was more commonly available in OM-WoID (79.1%) than in ABSSSI (43.4%), with MRSA emerging as the most frequently isolated pathogen in both groups (22.6% and 27.9%, respectively). Early discharge remained the primary reason for switching to DBV in both cohorts (67.9% in ABSSSI and 65.1% in OM-WoID), while treatment failure was reported in nearly one-third of cases. No adverse events were reported among OM-WoID patients receiving DBV. Results regarding the description of DBV use in ABSSSI and OM-WoID are extensively reported in Table 2.

Treatment success was achieved in 81.1% of ABSSSI cases and 90.7% of OM-WoID cases, with a comparable prevalence. No significant difference in clinical success was observed between the two MRSA syndromes (Table 2). All vertebral and skull base osteomyelitis were treated with success. In univariate analysis performed on the overall cohort, no covariates were significantly associated with treatment failure (Table 3). 

However, in the stepwise multivariate logistic regression, older age was independently associated with an increased risk of treatment failure (OR 1.07, 95% CI 1.01–1.13; *p* = 0.028), while the presence of multimorbidity significantly reduced the risk (OR 0.07, 95% CI 0.01–0.77; *p* = 0.029) (Table 4).

Candidate variables screened during the stepwise selection: age, sex, multimorbidity, renal function (eGFR), community-acquired infection, previous antibiotic therapy, polymicrobial infection, source control, reason to switch, concurrent antibiotic, adverse drug reactions.

## 3. Discussion

Our study highlights the extensive use of DBV in a real-world clinical setting, extending well beyond its current approved indication for ABSSSI. From 2017 to 2023, DBV prescriptions increased significantly during the study period, with off-label use accounting for 66.2% of cases—twice the frequency of on-label use (33.8%). Treatment success was achieved in 81.1% of ABSSSI cases and 90.7% of OM-WoID. These real-world data contribute to the growing body of evidence on off-label use of dalbavancin in selected clinical scenarios, suggesting a potential role in broader clinical settings, particularly enabling early patient discharge and optimizing healthcare resource utilization.

Intravenous antibiotic therapy is associated with high costs, intensive monitoring, and prolonged hospitalization [17]. In this context, DBV represents a valuable step-down option for invasive Gram-positive infections. Although evidence is currently based mainly on small-scale randomized controlled trials [18], single-center observational studies [19,20], and case series [21], support for its use is steadily increasing [17]. Beyond ABSSSI and osteomyelitis, growing evidence supports the off-label use of DBV in bloodstream infections and infective endocarditis [17]. Consistently, recent ESC guidelines suggest that, after adequate source control and an initial course of effective therapy, clinically stable patients may transition to oral therapy or OPAT, including long-acting lipoglycopeptides such as DBV [22,23].

In polymicrobial infections, which represented a minority of cases in our cohort, DBV was used as targeted therapy against Gram-positive pathogens within a broader antimicrobial regimen covering Gram-negative and/or anaerobic organisms, which likely contributed to overall clinical success. Similarly, the use of DBV as suppressive therapy represents a clinical application not originally anticipated in registrational studies. Suppressive therapy refers to the prolonged administration of antibiotics aimed at controlling infection and preventing relapse in cases where curative treatment is not feasible and, although the number of patients receiving DBV in a suppressive regimen within our cohort was limited (3.8%), our findings align with a growing body of literature supporting the role of DBV as a suppressive antibiotic therapy (SAT) in managing chronic infections, most often due to the inability to achieve adequate source control [24]. This approach is particularly relevant in complex infections such as prosthetic joint infections or prosthetic valve endocarditis, where surgical intervention may pose significant risks to the patient [24,25,26].

Moreover, while our cohort included only a limited number of pediatric patients (<18 years), emerging data also support the use of DBV in children and adolescents. Notably, a recent multicenter Italian study evaluated 31 pediatric patients treated with DBV for bone and joint infections as well as ABSSSI. The therapy demonstrated excellent tolerability, and no cases of treatment failure or adverse effects were reported during a median follow-up of two months. These findings suggest that DBV is not only safe and feasible in the pediatric population but also effective in reducing hospital length of stay [27].

In the stepwise multivariate logistic regression analysis of treatment failure risk in ABSSSI and OM-WoID, only older age was independently associated with increased failure risk, consistent with prior evidence in several settings of infectious diseases [28,29]. Conversely, multimorbidity was associated with a reduced risk, possibly reflecting residual confounding, selection bias and more intensive clinical management—these patients may receive closer monitoring, earlier intervention, and more tailored treatment, contributing to better outcomes [30]. This apparent protective effect may also reflect methodological artefacts, with few failures yielding unstable ORs and stepwise selection inflating potentially spurious associations; therefore, this association should be interpreted with caution and considered hypothesis-generating within the exploratory nature of this analysis.

Our results should be interpreted in light of several limitations. First, its retrospective design may introduce selection bias, particularly regarding missing data or inconsistencies in clinical documentation. Second, as a single-center study conducted in a tertiary care hospital, the generalizability of the findings to other clinical settings may be limited. The absence of a standardized protocol for DBV administration and follow-up reflects real-world practice but introduced heterogeneity in treatment regimens across off-label indications, limiting the feasibility of meaningful subgroup or regimen-specific analyses. Furthermore, some potential confounders that may influence treatment effectiveness, such as markers of infection severity, immunosuppression status and more granular comorbidity indices, were not systematically available and may therefore have contributed to residual confounding. In this context, the multivariable analysis, which was exploratory and restricted to patients with OM-WoID, should be interpreted with caution. Given the limited sample size and number of outcome events, the model may be subject to overfitting. The limited number of outcome events relative to the number of variables evaluated raises concerns regarding model stability and potential overfitting. This is particularly relevant to the observed protective association of multimorbidity, which lacks a clear biological explanation and is more likely attributable to residual confounding, patient selection, or statistical instability. Finally, although the comparison between ABSSSI and OM-WoID groups is informative, the observational nature of the study precludes definitive conclusions regarding efficacy, which would require randomized controlled trials. However, our study also presents several strengths. First, it offers a comprehensive real-world analysis of DBV use in both approved and off-label indications, including a substantial cohort of patients with OM-WoID, a population for which robust clinical data remain limited. The inclusion of microbiological, clinical, and therapeutic data enhances the clinical relevance of our findings and supports the growing evidence base for the off-label use of DBV, particularly in facilitating early discharge and outpatient management.

## 4. Materials and Methods

### 4.1. Study Design and Participants

This monocentric, retrospective study aims to describe the use of DBV with a particular focus on OM-WoID at ASST Spedali Civili di Brescia, Italy. All patients treated with DBV at this institution between April 2017 and July 2023 were included. Patients were excluded if demographic data were incomplete, or if case records lacked sufficient information due to the simultaneous absence of data on infection type, causative pathogen, treatment, or clinical outcome. The analysis focused on patients who received DBV for either ABSSSI or OM-WoID, with the latter group completing an appropriate course of intravenous antibiotic therapy prior to transitioning to DBV, in accordance with current local and international guidelines. The primary outcome was the treatment failure of DBV therapy, defined independently of on-label or off-label use as clinical deterioration during antimicrobial therapy, infection recurrence after completion of therapy or need for suppressive therapy.

### 4.2. Definitions

All diagnoses were primarily based on clinical assessment, with imaging and microbiological findings used as supportive evidence when available. ABSSSI were defined as bacterial infections involving the skin and associated soft tissues, including cellulitis, erysipelas, major cutaneous abscesses, and wound infections with significant extension into the dermis, subcutaneous tissue, or deeper anatomical structures [31]. OM-WoID was defined as infection of bone tissue diagnosed on a clinical basis and supported by imaging findings (e.g., radiography, computed tomography, or magnetic resonance imaging) and/or microbiological confirmation from bone or blood cultures when obtained [32]. Cases involving orthopedic or surgical implantable materials, including prosthetic joints, fixation plates, screws, or other internal hardware, were excluded from the OM-WoID category. Community-acquired infections (CAI) and hospital-acquired infections (HAI) were defined according to classical criteria, with CAIs identified within 48 h of hospital admission in patients without prior healthcare exposure, and HAIs defined as infections occurring more than 48 h after hospital admission [33]. For the purposes of this study, healthcare-associated (HCA) infections were defined using the modified criteria proposed by Cardoso et al., as infections identified within 48 h of admission in patients with prior contact with healthcare services within the preceding year [34]. Multimorbidity was defined as the concomitant presence of at least three comorbidities. Recurrence was defined as the reappearance of the same infectious syndrome within 60 days of treatment completion regardless of whether the causative microorganism was the same strain or a different one. Therefore, patients were followed for a minimum of 2 months after treatment completion; longer follow-up data were not consistently available. Conversely, “suppressive therapy” referred to the chronic administration of DBV in patients for whom neither adequate source control could be achieved nor a switch to oral antibiotic therapy was feasible. Clinical improvement for ABSSSI was defined as early reduction in lesion size and stabilization of systemic symptoms within 48–72 h, while for OM-WoID, it was defined by improvement in local symptoms and inflammatory markers over the first 1–2 weeks of therapy. “Worsening on treatment” was defined as either clinical deterioration during antimicrobial therapy, evidenced by progression of local or systemic signs of infection requiring a change or escalation of treatment. Treatment failure was defined as the occurrence of at least one of the following: recurrence, suppressive therapy, or worsening on treatment.

### 4.3. Data Collection

Patient medical charts were reviewed to collect relevant clinical data, including demographic information such as age and gender, as well as comorbidities, which were identified based on concomitant treatments. Additional variables included the type and site of infection, bacterial isolates along with the corresponding sample source, and details of antibiotic therapy administered prior to DBV initiation. Data on DBV treatment encompassed dosage, number of administered doses, and any reported adverse effects. Laboratory tests performed before DBV administration, at the end of treatment, and during follow-up were also recorded. All anonymized data were systematically recorded in a dedicated electronic database (REDCap) [35].

### 4.4. Ethics

The study protocol was approved by the Ethics Committee of ASST Spedali Civili (protocol n. 6293) and conducted in accordance with the ethical standards of the Helsinki Declaration (1975, revised in 2013). Informed consent was obtained from each subject.

### 4.5. Statistical Analysis

Categorical variables were expressed as counts and percentages, while continuous variables were summarized as means with standard deviations (SD) or medians with ranges (min–max), depending on their distribution. Comparisons of nonparametric continuous variables were conducted using the Mann–Whitney U test or the Kruskal–Wallis test, as appropriate, whereas categorical variables were analyzed using the χ^2^ test or Fisher’s exact test. For the inferential analysis, treatment failure was used as the dependent variable. Univariate binomial logistic regressions were conducted in patients treated with DBV for ABSSSI or OM-WoID. A multivariate logistic regression was conducted as an exploratory analysis restricted to patients with OM-WoID to identify independent predictors of treatment failure. Candidate variables were predefined based on clinical relevance and biological plausibility and entered into a stepwise selection process to limit model complexity. The final multivariate model included age, multimorbidity, reason for treatment switch, and concomitant antibiotic therapy. The number of covariates was deliberately limited in consideration of the sample size and number of outcome events. All statistical analyses were performed using R statistical software version 4.5.3 [36], with statistical significance set at an alpha level of <0.05.

## 5. Conclusions

Our analysis highlights the growing off-label use of DBV and the favourable clinical outcomes observed in OM-WoID. These findings add to the growing real-world evidence on the potential role of DBV beyond its current approved indications, particularly in facilitating early discharge and outpatient management. However, given the retrospective and uncontrolled design of the study, these results should be considered hypothesis-generating rather than confirmatory.

## Figures and Tables

**Table 1:** Demographic, Clinical, Microbiological, and Treatment Characteristics of Patients Treated with Dalbavancin. Acronyms used: DBV, dalbavancin; ABSSSI, acute bacterial skin and soft tissue infections; MSSA, methicillin-susceptible *Staphylococcus aureus*; MRSA, methicillin-resistant *Staphylococcus aureus*; MS-CoNS, methicillin-susceptible coagulase-negative staphylococci; MR-CoNS, methicillin-resistant coagulase-negative staphylococci; ADR, adverse drug reaction. * Success rate was calculated excluding cases with missing outcome or strain.

Sample, n (%)		157 (100)
Biographical		
Men, n (%)		99 (63.0)
Age, in years, mean (±SD)		62.5 (20)
Infection ward		
Medicine, n (%)		127 (80.9)
Surgery, n (%)		24 (15.3)
Pediatrics, n (%)		3 (1.9)
NA, n (%)		3 (1.9)
Most prevalent comorbidities		
Cardiovascular, n (%)		85 (54.6)
Diabetes, n (%)		44 (28.0)
None, n (%)		18 (11.5)
DBV prescription		
On-label indication, n (%)		53 (33.8)
Off-label indication, n (%)		104 (66.2)
Infection		
Community-acquired, n (%)		73 (46.5)
Healthcare-associated, n (%)		38 (24.2)
Hospital-acquired, n (%)		44 (28.0)
NA, n (%)		2 (1.3)
ABSSSI, n (%) Osteomyelitis, n (%)		53 (33.8) 60 (38.2)
Osteomyelitis without implantable devices, n (%)		43 (27.4)
Osteomyelitis with implantable devices, n (%)		17 (10.8)
Surgery site infections, n (%)		11 (7.0)
Prosthetic joint infections, n (%)		12 (7.6)
Endocarditis of native valve, n (%)		3 (1.9)
Endocarditis of prosthetic valve, n (%)		4 (2.6)
Vascular device infection, n (%)		6 (3.8)
Others, n (%)		8 (5.1)
Monomicrobial, n (%)		128 (81.5)
Polymicrobial, n (%)		24 (15.3)
Empirical therapy, n (%)		49 (31.2)
MSSA, n (%)		21 (13.4)
MRSA, n (%)		30 (19.1)
MS-CoNS, n (%)		9 (5.7)
MR-CoNS, n (%)		32 (20.4)
Other, n (%)		16 (10.2)
Isolation source (based on targeted treatment)		
Biopsy, n (%)		28 (25.9)
Intraoperative specimen, n (%)		25 (23.1)
Blood culture, n (%)		18 (16.7)
Lesion aspirate specimen, n (%)		14 (13.0)
Lesion swab, n (%)		23 (21.3)
Source control		
None, n (%)		113 (72.0)
Debridement/toilette, n (%)		23 (14.7)
Of which hospitalized in a surgical ward, n (%)		9 (5.4)
Hardware removal, n (%)		20 (12.7)
Of which hospitalized in a surgical ward, n (%)		6 (3.6)
NA, n (%)		1 (0.6)
Reason to switch		
Early discharge, n (%)		104 (66.3)
Adverse effect/intolerance, n (%)		6 (3.8)
Unspecified or unknown, n (%)		47 (29.9)
Outcome		
Clinical improvement or recovery, n (%)		121 (77.1)
Treatment failure, n (%)		28 (17.8)
Including	Worsening on treatment, n (%)	12 (7.6)
	Recurrence, n (%)	10 (6.4)
	Suppressive therapy, n (%)	6 (3.8)
Recurrence, not from Gram+, n (%)		4 (2.6)
Lost to FU, n (%)		4 (2.6)
Clinical improvement according to etiological agent *		
MSSA, n/N (%)		14/20 (70)
MRSA, n/N (%)		23/31 (74)
MS-CoNS, n/N (%)		8/10 (80)
MR-CoNS, n/N (%)		24/32 (75)
ADR		
None, n (%)		149 (94.9)
Allergy, n (%)		2 (1.3)
Yes but not known, n (%)		1 (0.6)
NA, n (%)		5 (3.2)

**Table 2:** Characteristics and Demographics of the Study Sample (Acronyms used: ABSSSI, acute bacterial skin and soft tissue infections; OM-WoID, osteomyelitis without implantable devices; DBV, dalbavancin; MSSA, methicillin-susceptible *Staphylococcus aureus*; MRSA, methicillin-resistant *Staphylococcus aureus*; MS-CoNS, methicillin-susceptible coagulase-negative staphylococci; MR-CoNS, methicillin-resistant coagulase-negative staphylococci; GP, Gram-positive; GN, Gram-negative; IV, intravenous; ADR, adverse drug reaction). * Success rate was calculated excluding cases with missing outcome or strain.

		ABSSSI	OM-WoID	*p*-Value
N (%)		53 (100)	43 (100)	
Biographical				
Men, n (%)		26 (49.1)	29 (67.4)	0.097
Age, in years, median (range)		63 (10–91)	70 (9–92)	0.874
Infection ward				0.704
Medicine, n (%)		45 (84.9)	37 (86.1)	
Surgery, n (%)		2 (3.8)	4 (9.3)	
Pediatrics, n (%)		1 (1.9)	1 (2.3)	
NA, n (%)		5 (9.4)	1 (2.3)	
Infection setting				0.061
Community-acquired, n (%)		34 (64.2)	17 (39.5)	
Healthcare-associated, n (%)		9 (16.4)	20 (46.5)	
Hospital-acquired, n (%)		9 (16.4)	6 (14.0)	
NA, n (%)		1 (1.9)	0 (0)	
Comorbidities (more prevalent)				
Cardiovascular, n (%)		25 (58.1)	16 (37.2)	
Diabetes, n (%)		13 (24.5)	8 (18.6)	
Solid neoplasm, n (%)		9 (17.0)	4 (9.3)	
Trauma, n (%)		9 (17.0)	6 (14.0)	
None, n (%)		6 (11.3)	5 (11.6)	
Isolations, n (% out of all included patients)				
MSSA, n (%)		4 (7.6)	8 (18.6)	
MRSA, n (%)		12 (22.6)	12 (27.9)	
MS-CoNS, n (%)		0 (0.0)	3 (7.0)	
MR-CoNS, n (%)		5 (9.4)	8 (18.6)	
Other, n (%)		4 (7.6)	15 (35)	
Empirical therapy, n (%)		30 (56.6)	9 (20.9)	
Polymicrobial, n (%)		5 (9.4)	5 (11.6)	
Prior antibiotic therapy				0.062
Anti-GP, n (%)		17 (32.1)	3 (7.0)	
Anti-MRSA IV, n (%)		4 (7.6)	5 (11.6)	
Anti-MRSA oral, n (%)		5 (9.4)	5 (11.6)	
Anti-GN IV + anti-MRSA IV, n (%)		9 (17.0)	18 (41.9)	
Anti-GN oral + anti-MRSA oral, n (%)		5 (9.4)	4 (9.3)	
Anti-GN, n (%)		3 (5.7)	2 (4.7)	
NA or unspecified, n (%)		10 (18.9)	6 (14.0)	
Outcome				
Clinical improvement or recovery, n (%)		43 (81.1)	39 (90.7)	0.28
Treatment failure, n (%)		7 (13.2)	3 (6.9)	0.51
Including	Worsening on treatment, n (%)	5 (9.4)	1 (2.3)	
	Recurrence, n (%)	2 (3.8)	1 (2.3)	
	Suppressive therapy, n (%)	0 (0)	1 (2.3)	
Recurrence, not from Gram+, n (%)		0 (0)	1 (2.3)	
Lost to FU, n (%)		3 (5.7)	0 (0)	
Clinical improvement according to etiological agent *				
MSSA, n/N (%)		3/4 (75)	5/7 (71.4)	1
MRSA, n/N (%)		8/10 (80)	12/12 (100)	0.195
MS-CoNS, n/N (%)		/	3/3 (100)	1
MR-CoNS, n/N (%)		5/5 (100)	7/8 (87.5)	1
Reason to switch to DBV				0.542
Therapy failure with prior antibiotic, n (%)		16 (30.2)	12 (27.9)	
Early discharge, n (%)		36 (67.9)	28 (65.1)	
ADR at prior therapy, n (%)		1 (1.9)	2 (4.7)	
NA, n (%)		0 (0.0)	1 (2.3)	

**Table 3:** Univariate Logistic Regressions for treatment failure as outcome (Acronyms used: OR, odds ratio; eGFR, estimated glomerular filtration rate; ADR, adverse drug reactions).

Covariate	OR (95% Confidence Interval)	*p*-Value
On-label prescription	2.59 (0.61–11.04)	0.198
Age (years)	1.03 (0.99–1.08)	0.145
Female	1.59 (0.41–6.13)	0.500
Multimorbidity	0.15 (0.02–1.29)	0.084
eGFR (mL/min/1.73 m^2^)	0.67 (0.31–1.47)	0.318
Community-acquired infection	1.09 (0.45–2.67)	0.842
Previous antibiotic therapy	0.38 (0.06–2.33)	0.298
Polymicrobial infection	0.52 (0.06–4.60)	0.558
Source control	0.65 (0.09–4.90)	0.673
Reason to switch	0.36 (0.09–1.37)	0.133
Concurrent antibiotics	0.28 (0.05–1.42)	0.125
ADR	0.00 (0.00–Inf)	0.995

**Table 4:** Stepwise Multivariate Logistic regression (Acronyms used: OR, odds ratio; CI, confidence interval).

Variable	OR	CI Low (95%)	CI Up (95%)	*p*-Value
On-label prescription	2.95168873	0.5382696531	16.1860627	0.21253785
Age (years)	1.06817997	1.0069854469	1.1330933	0.02843215
Multimorbidity	0.07271259	0.0069102601	0.7651116	0.02903814
Reason to switch	0.23619135	0.0443239980	1.2586039	0.09092105
Concurrent antibiotic therapy	0.22681939	0.0312888602	1.6442605	0.14211670

## Data Availability

The data presented in this study are available on request from the corresponding author.
